# Supplementary material for: Possible risk factors for poor asthma control assessed in a cross-sectional population-based study from Telemark, Norway
Source: PLoS One. 2020 May 12;15(5):e0232621. doi: 10.1371/journal.pone.0232621 (PMC7217450; doi:10.1371/journal.pone.0232621)
Supplement: S1 File — (PDF) [file pone.0232621.s001.pdf]

Dato:

ID nummer:

Alder:

Kjønn:

□ Kvinne

□ Mann

Målt vektt: kg

Målt høyde: cm

1. Har du blitt undersøkt med spirometri noen gang før?

□ No

□ Yes

2. Bruker du for tiden medisin mot astma?

□ No

□ Yes

3. Har du vært hos lungelege noen gang?

□ No

□ Yes

4. Har en lege noen gang gitt deg diagnosen astma?

□ No

□ Yes

5. Har du i ditt arbeid de siste 12 månedene vært utsatt for gass, støv, røyk eller damp?

□ Nei

□ Ja

6. Har du allergi som gir symptomer fra nesen, inkludert høysnue?

□ Nei

□ Ja

7. Har du hatt astma anfall de siste 12 månedene?

□ Nei

□ Ja

8. Har du våknet av anfall med tungpust på noe tidspunkt i løpet av de siste 12 månedene?

□ Nei

□ Ja

9. Har du noen gang hatt piping eller hvesing i brystet i løpet av de siste 12 månedene?

□ Nei

□ Ja

10. Har du på noe tidspunkt de siste 12 månedene oppsøkt fastlege, legevakt eller akuttmottak på grunn av akutt pustebesvær?

11. Har du på noe tidspunkt de siste 12 månedene vært innlagt på sykehus på grunn av pustebesvær?

□ Nei

□ Ja

12. Har du tatt ekstra kortisonmedisiner eller økt din kortisoninhalasjon på noe tidspunkt de siste 12 månedene?

□ Nei

□ Ja

13. Røyker du daglig (gjelder selv om du kun røyker noen få sigaretter, sigarer eller pipe daglig)?

□ Nei

□ Ja

Røyker du bare av og til (ikke daglig, men helger, festrøyking eller liknende)?

□ Nei

□ Ja

Har du røykt tidligere?

□ Nei

□ Ja

Hvis bare NEI-svar på røyking trenger du ikke å fylle ut resten av spørsmålene.

Hvor gammel var du da du begynte å røyke? År

Hvor lenge har du røykt (gjelder både nåværende og tidligere røyking)? År

Tusen takk for dine svar!
