# Supplementary material for: Possible risk factors for poor asthma control assessed in a cross-sectional population-based study from Telemark, Norway
Source: PLoS One. 2020 May 12;15(5):e0232621. doi: 10.1371/journal.pone.0232621 (PMC7217450; doi:10.1371/journal.pone.0232621)
Supplement: S2 File — (PDF) [file pone.0232621.s002.pdf]

Date:

ID number:

Age:

Gender:

□ Female

□ Male

Measured weight: kg

Measured height: cm

1. Have you ever been examined by spirometry?

□ No

□ Yes

2. Do you currently use medication for asthma?

□ No

□ Yes

3. Have you ever visited a pulmonary physician?

□ No

□ Yes

4. Has a physician ever diagnosed you with asthma?

□ No

□ Yes

5. Have you in your work been exposed to: vapor, gas, dust, or fumes during the past 12 months?

□ No

□ Yes

6. Do you suffer from any allergy that gives symptoms from the nose (including hey fever)?

□ No

□ Yes

7. Have you experienced an asthma attack during the past 12 months?

□ No

□ Yes

8. Have you been awakened by heavy breathing/dyspnea at any time during the past 12 months?

□ No

□ Yes

9. Have you experienced whistling or wheezing in your chest at any time during the past 12 months?

□ No

□ Yes

10. Have you visited a doctor or accident/emergency unit because of acute breathing difficulties at any time in the past 12 months?

□ No

□ Yes

11. Have you been hospitalized because of breathing difficulties at any time during the past 12 months?

□ No

□ Yes

12. Have you used extra cortisone medication or increased your cortisone inhalation at any time during the past 12 months?

□ No

□ Yes

13. Do you smoke (also if a few cigarettes, sigars or pipe) every day?

□ Nei

□ Ja

Do you smoke occationally?

□ Nei

□ Ja

Have you smoked previously?

□ Nei

□ Ja

If you have never smokes, you don't need to answer the following questions

How old were you when you started smoking? Years

How many years have you smoked altogether? Years
