# Supplementary material for: Possible risk factors for poor asthma control assessed in a cross-sectional population-based study from Telemark, Norway
Source: PLoS One. 2020 May 12;15(5):e0232621. doi: 10.1371/journal.pone.0232621 (PMC7217450; doi:10.1371/journal.pone.0232621)
Supplement: Attachment — Submitted filename: Response to Reviewers.docx [file pone.0232621.s003.docx]

Point to point response to editors and reviewers’ comments:

*First, we would like to thank the reviewer for the thorough review and valuable comments that have allowed us to adjust accordingly, and improve the manuscript significantly. Authors comments are in italic.*

**Journal Requirements:**

When submitting your revision, we need you to address these additional requirements.

1. Please ensure that your manuscript meets PLOS ONE's style requirements, including those for file naming. The PLOS ONE style templates can be found at http://www.journals.plos.org/plosone/s/file?id=wjVg/PLOSOne_formatting_sample_main_body.pdf and http://www.journals.plos.org/plosone/s/file?id=ba62/PLOSOne_formatting_sample_title_authors_affiliations.pdf

*We have now labeled the files according to the style template.*

2. Please include additional information regarding the survey or questionnaire used in the study and ensure that you have provided sufficient details that others could replicate the analyses. For instance, if you developed a questionnaire as part of this study and it is not under a copyright more restrictive than CC-BY, please include a copy, in both the original language and English, as Supporting Information.

*We have now included both a Norwegian and an English copy of the questionnaire as Supporting Information.*

3. Thank you for stating the following financial disclosure:

"The funders had no role in study design, data collection and analysis, decision to publish, or preparation of the manuscript."

a.	Please provide an amended Funding Statement that declares *all* the funding or sources of support received during this specific study (whether external or internal to your organization) as detailed online in our guide for authors at http://journals.plos.org/plosone/s/submit-now.

*This is now included in the cover letter.*

b.	Please state what role the funders took in the study. If any authors received a salary from any of your funders, please state which authors and which funder. If the funders had no role, please state: "The funders had no role in study design, data collection and analysis, decision to publish, or preparation of the manuscript." Please include your amended statements within your cover letter; we will change the online submission form on your behalf.

*This is now included in the cover letter.*

4. In your Data Availability statement, you have not specified where the minimal data set underlying the results described in your manuscript can be found. PLOS defines a study's minimal data set as the underlying data used to reach the conclusions drawn in the manuscript and any additional data required to replicate the reported study findings in their entirety. All PLOS journals require that the minimal data set be made fully available. For more information about our data policy, please see <http://journals.plos.org/plosone/s/data-availability>. Upon re-submitting your revised manuscript, please upload your study’s minimal underlying data set as either Supporting Information files or to a stable, public repository and include the relevant URLs, DOIs, or accession numbers within your revised cover letter. For a list of acceptable repositories, please see http://journals.plos.org/plosone/s/data-availability#loc-recommended-repositories. Any potentially identifying patient information must be fully anonymized. Important: If there are ethical or legal restrictions to sharing your data publicly, please explain these restrictions in detail. Please see our guidelines for more information on what we consider unacceptable restrictions to publicly sharing data: http://journals.plos.org/plosone/s/data-availability#loc-unacceptable-data-access-restrictions. Note that it is not acceptable for the authors to be the sole named individuals responsible for ensuring data access. We will update your Data Availability statement to reflect the information you provide in your cover letter.

*This is also included in the cover letter.*

**5. Review Comments to the Author**

Reviewer #1: This was a very interesting study, which was well-written. Here are my specific observations and suggestions:

1. Line 38: how likely is it that the youngest participants were exposed to occupational fumes? It would be helpful to provide an additional table stratifying the descriptive statistics by age category due to heterogeneity in the sample.

*We have performed the stratification on age for the results in table 1. We have chosen not to include these in a new table because the table would be considerably larger and the important and interesting results are the following: “In the age group, 16-30; 36% of participants reported exposure to VGDF, while the percentage for those aged 31-40 and 41-50 were 27% and 24%, respectively.” This sentence is now included in the results section.*

2. Sensitivity analysis conducted by age group. Stratify the analysis by age groups due to the different stages of lung development across the age range. It is possible that some older participants have COPD, for example, and thus poorer lung function. Furthermore, teenagers are likely to under/mis-report smoking habits.

*As suggested, we have performed sensitivity analyses (stratification) by age. However, the groups were small and the confidence intervals overlapping (results not shown) and thus to our opinion no meaningful information could be derived from these analyses. However, we have included the following to the statistic section: “ Further, stratification by age was performed but the groups were small and the confidence intervals overlapping (results not shown).”*

*In addition, we added the following regarding COPD and young participants (regarding the latter a new reference was added nr. 42) to the limitation section of the discussion:*

1. *“To reduce the probability of chronic obstructive pulmonary disease (COPD), we restricted the study group to ≤50 years.”*
2. *“We restricted the inclusion of participants to the age group 16 to 50 years. The youngest age group (16 to 30 years) reported the highest prevalence of occupational VGDF exposure (36%). Although a substantial proportion of these subjects were unexposed students, apprentices and young workers may have a higher probability of exposure [42].”*

3. Education, smoking, obesity and possibly VGDF are likely to be proxies for socio-economic status and all correlated. The authors should investigate multicollinearity in their models and the relationships between these variables before including them in the regression models.

*The following has been included in the statistics section: “Collinearity was investigated by Pearson correlation showing weak correlations between the included proxies for socioeconomic status (education, smoking, obesity and VGDF). The strongest correlation was between education and smoking (r=0.157).”*

*Because income is not necessarily a good measure of socioeconomic status in Scandinavia, education is often used. We have choose also to adjust for smoking because of the association with asthma severity. We have shown in other studies (based on the same cohort) that asthma is associated with obesity and exposure to VGDF. Hence, the results were adjusted for these four variables.*

4. Line 164: “adjustment obesity” - please add “for”.

*Thank you, this has been corrected.*

5. Table 1: Poorly controlled asthma for age: %s do not add to 100.

*This is due to rounding of numbers.*

6. What are the reference/normal ranges for the clinical variables? This would provide greater clarity about differences and severity between both groups.

*The reference values/range are now included in table 2.*

7. Greater clinical context is required about what the clinical variables mean and why they are relevant. To reader with limited clinical knowledge, it isn’t clear what the measures imply given the results. For example, there is no discussion about what CRP is, why it has been included in the study, and what the results mean clinically.

*We have met this comment by inclusion of the following information in the methods section (the new text is underlined) : Measurement of the concentration of IgE was included to assess allergic response and analyzed using a Siemens Immulite 2000 XPI at the Department of Laboratory Medicine, Telemark Hospital, Skien. High-sensitivity CRP was included as a marker of systemic inflammation and analyzed using a Modul c702 Cobas 8000 modular analyzer (Roche Diagnostics) at the Department of Medical Biochemistry, Oslo University Hospital (Ullevål), Oslo.*

*Accordingly, the following adjustment was made regarding FeNO: The fraction of exhaled nitric oxide (FeNO) in exhaled air was included as a marker of eosinophilic inflammation, and was measured according to the ATS/ERS criteria using a NIOX MINO (Aerocrine AB, Solna, Sweden) [13].*

*In addition, the following adjustment (the new text is underlined) is made to the corresponding text in the discussion section: Table 1 shows that poor asthma control was associated with both an elevated level of the systemic inflammatory marker CRP and a reduced level of FeNO. After adjusting for possible confounders, CRP and FeNO were no longer significant (Table 4). This may imply that the univariate association is due to the confounders. Alternatively, the inclusion of not only severe asthma patients with signs of systemic inflammation or allergic response, but the whole range of subjects who had reported asthma symptoms in the previous 12 months could explain this finding.*

8. Table 4 and Methods: This is very confusing and unclear. I thought that poor asthma control (a binary variable) is the outcome throughout the paper (as also implied by the title of Table 4). If this is the case, your model is incorrect. Linear regression is for continuous outcomes. In light of these comments, the analysis and discussion surrounding Table 4 is unclear. Clarity is required on why you have chosen the model, what is the hypothesised causal relationship, and which variables are the dependent/independent variables.

*Thank you for this important comment. We agree that the table title and text was unclear. Linear regression was used to identify differences in clinical variables between poor and well-controlled asthma cases. The clinical variables were the dependent in these analyses. We have now changed the title of table 4 and the text referring to this table in the results and discussion sections accordingly.*

9. The median and IQR has been reported for some clinical variables, suggesting they are not normally distributed. The authors should check whether the assumptions of the linear regression model have been upheld and whether these variables require transformation (e.g., logged).

*We have now performed the analyses on log transformed variables for IgE, CRP and FeNO (table 4). The conclusions are upheld, but we agree that the log transformed variables should be used and have included them in table 4.*

10. Line 249: The authors should investigate whether non-significance of clinical variables could be due to the distribution of these variables.

*Log transformed variables for IgE, CRP and FeNO are now used and did slightly influence the numbers, but the results are still not statistical significant (table 4). Please see also our response to comment 9.*

11. Results have been presented for the first time in the Discussion section. I suggest that these should be presented in the Results section.

*Thank you, we have now also included the following to the text in the results section: “Twenty-four percent of participants with asthma symptoms during the previous 12 months, and 13% of those with poorly controlled asthma had not used asthma medication.”*

*On behalf of the authors,*

*Sincerely yours*

*Anne Kristin M. Fell, MD, PhD*

*Corresponding author*
